# Outbreak of Hantavirus Pulmonary Syndrome, Los Santos, Panama, 1999–2000

**DOI:** 10.3201/eid1009.040143

**Published:** 2004-09

**Authors:** Vicente Bayard, Paul T. Kitsutani, Eduardo O. Barria, Luis A. Ruedas, David S. Tinnin, Carlos Muñoz, Itza B. de Mosca, Gladys Guerrero, Rudick Kant, Arsenio Garcia, Lorenzo Caceres, Fernando G. Gracia, Evelia Quiroz, Zoila de Castillo, Blas Armien, Marlo Libel, James N. Mills, Ali S. Khan, Stuart T. Nichol, Pierre E. Rollin, Thomas G. Ksiazek, Clarence J. Peters

**Affiliations:** * Gorgas Memorial Institute for Health Studies, Panama City, Panama;; † University of Panama, Panama City, Panama;; ‡ Centers for Disease Control and Prevention, Atlanta, Georgia, USA;; § University of Wisconsin, Madison, Wisconsin, USA;; ¶ University of New Mexico, Albuquerque, New Mexico, USA;; # Ministry of Health, Panama City, Panama;; ** Complejo Hospital A.A.M., Panama City, Panama;; †† Pan American Health Organization, Washington DC, USA;; ‡‡ University of Texas, Galveston, Texas, USA

**Keywords:** Hantavirus pulmonary syndrome, hantavirus, kidney diseases, biliary disease, hemorrhage, *Zygodontomys*, *Oligoryzomys fulvescens*, Panama, research

## Abstract

An outbreak of hantavirus pulmonary syndrome occurred in the province of Los Santos, Panama, in late 1999 and early 2000. Eleven cases were identified; 9 were confirmed by serology. Three cases were fatal; however, no confirmed case-patient died. Case-neighborhood serologic surveys resulted in an overall hantavirus antibody prevalence of 13% among household and neighborhood members from the outbreak foci. Epidemiologic investigations did not suggest person-to-person transmission of hantavirus infection. By use of Sin Nombre virus antigen, hantavirus antibodies were detected in *Oligoryzomys fulvescens* and *Zygodontomys brevicauda cherriei*. This outbreak resulted in the first documented cases of human hantavirus infections in Central America.

## Abstract 2

The first identified outbreak of hantavirus pulmonary syndrome in Central America is described.

Hantavirus pulmonary syndrome (HPS) is an infectious disease typically characterized by fever, myalgia, and headache and followed by dyspnea, noncardiogenic pulmonary edema, hypotension, and shock (*1**,**2*). Common laboratory findings include elevated hematocrit, leukocytosis with the presence of immunoblasts, and thrombocytopenia (*3**,**4*). The case-fatality rate can be as high as 52% (*5*). The etiologic agent of HPS is any one of several hantaviruses carried by rodent hosts belonging to the family *Muridae*, subfamily Sigmodontinae (*6*). Hantaviruses are most often transmitted to humans through the inhalation of infectious rodent feces, urine, or saliva. However, strain-specific virus transmission may occur from person to person (*7**–**9*).

HPS was first recognized in 1993 during an outbreak of severe respiratory disease in the Four Corners Region of the United States (*10**,**11*). Since then, 363 cases of HPS have been confirmed in the United States (*12*). Sin Nombre virus (SNV) was the first HPS-causing pathogen identified; its primary rodent reservoir host is the deer mouse, *Peromyscus maniculatus* (*13**,**14*). However, four other hantaviruses, Bayou virus, Black Creek Canal virus, New York virus, and Monongahela virus, each with a different rodent reservoir, have been characterized in the United States and associated with HPS (*6**,**15**–**21*).

Since 1993, HPS has also been reported and confirmed in six countries in South America—Argentina, Bolivia, Brazil, Chile, Paraguay, and Uruguay (*6**,**9**,**22**–**30*). Several distinct hantaviruses have been associated with HPS, including Andes virus (Argentina, Bolivia, Chile, and Uruguay), Bermejo virus (Bolivia), Juquitiba virus (Brazil), Laguna Negra virus (Bolivia, Paraguay), Lechiguanas virus (Argentina), and Oran virus (Argentina) (*22**,**23**,**25**,**28**,**30**,**31*).

Before 2000, no human hantavirus infections had been reported in Central America. However, in February 2000, health officials in Panama reported a cluster of acute respiratory illnesses in residents of the district of Las Tablas in Los Santos Province from late December 1999 to February 2000 (*32*). Human illness was characterized by a febrile prodrome with rapid progression to moderate-to-severe respiratory distress. Serum specimens from three of four patients had immunoglobulin (Ig) M and IgG antibodies to SNV. In February and March 2000, an outbreak investigation by the Panamanian Ministry of Health, Gorgas Memorial Institute for Health Studies (Panama City), the U.S. Centers for Disease Control and Prevention (Atlanta, GA), and the Pan American Health Organization was conducted in collaboration with local medical and public health officials. Sequence analysis of virus genome from human serum samples and rodent tissue led to the identification of a novel hantavirus, Choclo virus, as the cause of HPS in this outbreak (*33*). This report summarizes the clinical, epidemiologic, and environmental findings of the investigation.

## Materials and Methods

### Case Definition

A suspected case of HPS was defined as fever (temperature >38.3°C) and unexplained acute respiratory distress requiring supplemental oxygen, with radiographic evidence of acute respiratory distress syndrome or bilateral interstitial pulmonary infiltrates (*34*). A suspected case was also defined as an unexplained respiratory illness resulting in death, with a postmortem examination indicating noncardiogenic pulmonary edema without identifiable cause (*34*). A confirmed case of HPS was defined as a clinically compatible illness plus the presence of one of the following; 1) hantavirus-specific IgM antibodies in acute-phase serum, 2) hantavirus-specific nucleic acid sequences by reverse transcriptase polymerase chain reaction (RT-PCR), or 3) hantavirus-specific antigen by immunohistochemistry (*34*).

### Case Finding and Characterization

At visits to two hospitals in Panama City and one in the Las Tablas District that admitted patients with suspected HPS, hospitalized patients were examined and interviewed when possible, and medical charts were reviewed. To identify past HPS patients, retrospective chart reviews were conducted on admissions dating back to August 1999 at these hospitals. In addition, medical records of any previous or current suspected HPS patients admitted to other district hospitals in the Las Tablas Province were obtained and reviewed. Clinical case information was collected on a standard abstraction form.

To monitor the spread of disease in Las Tablas and other areas of Los Santos Province, an outbreak communications center was established at the Ministry of Health in Panama City and staffed by physicians, public health officials, and health educators. Operations of this center included 1) passive surveillance for suspected cases of HPS, 2) a public hotline that addressed symptoms and signs of HPS and methods of prevention, and 3) nationwide distribution of HPS educational materials. Staff physicians also called hospitals throughout Panama to promote awareness of HPS among medical providers.

### Community Surveys

Surveys for hantavirus antibodies were conducted on March 5, 2000, in six neighborhoods in the Las Tablas District and one in the Guarare District in which confirmed HPS patients resided. The primary objectives of the surveys were to determine the prevalence of hantavirus infection within households and neighborhoods of case-patients and the frequency of mild and asymptomatic infection.

Approximately 10–15 households, including the case-patient household, were sampled in each of the seven neighborhoods. Teams composed of public health officials, nurses, and phlebotomists visited each neighborhood. The day before the survey, pamphlets explaining the survey in Spanish were given to each household that had agreed to participate. All participants were administered a standard questionnaire that asked about demographic characteristics, illness history, and rodent exposure. Team members were familiarized with the questionnaire before the survey started. A 10-mL blood specimen (3–5 mL for children) for hantavirus serologic testing was collected from each interviewed participant.

### Hospital Survey

A hospital survey was conducted March 9–10, 2000, among healthcare workers who cared for confirmed HPS patients at a major hospital in Panama City. This hospital received most of its cases from Las Tablas. The objectives of the survey were to 1) serologically determine exposure to hantavirus among doctors, nurses, respiratory therapists, physiotherapists, and nurses' aides who provided direct care to confirmed HPS patients and 2) assess whether person-to-person transmission occurred. Medical staff who provided direct medical care (i.e., <1 m from the patient) to infected patients in the emergency room and medical intensive care unit were compared with those in the coronary or neurologic intensive care units who had no exposure to case-patients. A standard self-administered questionnaire was used to inquire about timing and amount of exposures, specific types of exposures, and precautionary measures taken. A 10-mL blood specimen for hantavirus serologic testing was collected from each surveyed participant.

### Rodent Investigation

Small mammals were sampled to determine potential hantavirus reservoir hosts. Primary sampling methods were trapping and collecting small mammals around households of confirmed and suspected HPS patients and from two uninhabited locations in Los Santos. The habitat of each trapping area was described. Small mammals were initially identified in the field on the basis of external characteristics and standard keys for the region. Definitive identification that used cranial characteristics was performed at the Museum of Southwest Biology, University of New Mexico, where voucher specimens from these small mammals are currently archived. Liver, kidney, spleen, lung, heart, and a whole blood sample were collected from each trapped rodent for diagnostic testing, and carcasses were preserved for rodent speciation. Trapping and sampling were performed according to established safety protocols (*35*). Panamanian team members were trained to trap and observe safety precautions when handling potentially infectious rodents.

### Diagnostic Testing

All available serum specimens from suspected HPS patients were tested for IgM antibodies by using an IgM-capture format and inactivated SNV antigen and for IgG antibodies by an indirect enzyme-linked immunosorbent assay (ELISA) as previously described (*11*). Serum samples from survey participants, rodents, and other small mammals were tested for IgG antibodies by using SNV antigens. IgG-positive survey participants were also tested for IgM antibodies by use of SNV antigens. Positive findings with SNV antigens in the IgG- or IgM-capture ELISAs indicate infections with New World hantaviruses rather than with SNV specifically.

## Results

### Characteristics of HPS Patients

**Figure 1:**
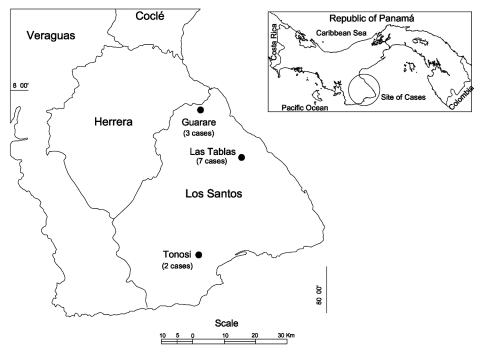
Location of districts in Los Santos Province, Republic of Panama, in which hantavirus cases occurred in 1999 and 2000.

Eleven case-patients with suspected HPS were identified and reported to the Panamanian Ministry of Health from December 25, 1999, to February 29, 2000 (Figure 1). Three patients (25%) died; neither serum nor tissue samples were available for diagnostic testing from these three patients. The remaining eight cases were confirmed by the presence of IgM against hantavirus in at least two separate serum specimens from patients; all eight also had IgG antibodies, with five demonstrating IgG seroconversion after the first specimen. One additional patient, whose onset of illness was August 24, 1999, was identified retrospectively. Although acute-phase clinical specimens were not available for testing, review of medical records indicated that her illness was clinically compatible with HPS, and her IgG titer in February 2000 was elevated (6,400). She was subsequently confirmed as the ninth HPS patient.

The median age of the 12 patients was 42 years (range 26–58 years); seven (58%) were women. The primary occupation of patients was as follows: secretary (2), housewife (2), truck driver/transporter (2), electrician (1), field pump worker (1), seamstress (1), teacher (1), security guard (1), and unidentified (1).

All cases occurred in the Las Tablas, Guarare, and Tonosi Districts of Los Santos Province. In Las Tablas and Guarare, eight towns had at least one patient with suspected HPS, and seven had one or more confirmed patients (Table 1). Two cases, one suspected and one confirmed, were identified in Tonosi. No clustering of cases occurred at the household level.

**Table 1:** Geographic distribution of suspected and confirmed HPS cases, Los Santos Province, Panama, August 1999–February 2000^a^

Town	District	No. confirmed cases	No. suspected cases
El Cocal	Las Tablas	1	0
La Tronosa	Las Tablas	0	1
Las Tablas	Las Tablas	1	0
Los Angeles	Las Tablas	1	0
San Jose	Las Tablas	2	0
Santo Domingo	Las Tablas	1	0
Bella Vista	Guarare	1	0
La Enea	Guarare	1	1
Las Llanas	Tonosi	1	1

^a^HPS, hantavirus pulmonary syndrome.

Two patients denied handling or cleaning up rodent excreta before their illnesses, while a third admitted frequently killing and handling rodents. In interviews, family members of all case-patients from Las Tablas District noted marked increases in the number of rodents in and around the home from November 1999 to February 2000.

### Clinical Description of Confirmed HPS Patients

The spectrum of preadmission symptoms of the nine confirmed patients is documented in Table 2. The mean temperature on admission was 38.0°C (n = 6, range 37.3°C–39.6°C). The median systolic and diastolic blood pressure values were 100 mm Hg (n = 9, range 80–130) and 63 mm Hg (n = 9, range 50–80), respectively. The median pulse rate was 102/min (n = 8, range 60–172). The median respiratory rate was 23/min (n = 8, range 14–36). No patient was unconscious or cyanotic on admission. A temperature of >38.5°C was documented in all patients at some time during hospitalization. Seven of nine patients were hypotensive during their illness (median lowest systolic blood pressure = 90, range 50–100), and three required inotropic therapy.

**Table 2:** Symptoms on admission in nine patients with laboratory-confirmed hantavirus pulmonary syndrome, Los Santos Province, Panama, August 1999–February 2000

Symptom	No. (%)
Fever	9 (100)
Dyspnea	8 ( 89)
Myalgia	8 ( 89)
Cough	7 ( 78)
Malaise	6 ( 67)
Vomiting	5 ( 56)
Nausea	4 ( 44)
Headache	4 ( 44)
Arthralgia	4 ( 44)
Weakness	4 ( 44)
Lower back pain	3 ( 33)
Abdominal pain	2 ( 22)
Chest pain	2 ( 22)
Dizziness	2 ( 22)
Diarrhea	1 ( 11)
Dysuria	1 ( 11)
Lower extremity edema	1 ( 11)

Laboratory values at admission and during hospitalization are listed in Table 3. Eight of nine patients were thrombocytopenic either on admission or at some time during hospitalization. Five of nine had hematuria (urine erythroctyes >10 per high power field, while four of nine had moderate proteinuria (<2+ on urine dipstick). Evidence of renal function abnormalities was present in three of nine patients whose serum creatinine levels were >2.0 mg/dL.

**Table 3:** Laboratory values of confirmed HPS patients on admission and during hospitalization^a^

Test	No. of cases	Median value (admission)	Range	No. of cases	Median maximum value (admission + hospitalization)	Range	Median minimum value (admission + hospitalization)	Range
Leukocyte count (x1,000/mm^3^)	7	6.4	3.6–14.1	9	11.4	5.7–28.1		
Hematocrit (%)	7	39.7	35.1–44.2	9	41.0	31.4–52.4		
Platelet (x1,000/mm^3^)	7	84	47–186	9			60	26–429
BUN (mg/dL)	2	7	5–9	8	26	1–56		
Creatinine (mg/dL)	3	0.8	0.6–1.0	8	2.2	1.0–3.3		
CPK (IU/L)				6	240	38–12,840		
LDH (U/L)	1	219	219^b^	7	1028	32–3,775		
Albumin (g/dL)				7			2.5	1.2–3.2
AST (U/L)	2	42	40–43	7	223	46–983		
ALT (U/L)	2	33	31–35	7	168	32–570		
Bilirubin, total (mg/dL)				7	1.1	0.8–8.0		
Bilirubin, direct (mg/dL)				7	0.6	0.1–6.5		
Amylase (U/L)				6	74	38–437		
PT (s)	1	11.7	11.7–11.7	4	13.7	11.3–22.0		
PTT (s)	1	31.2	31.2^b^	4	28	27–167		
Urine leukocyte count (/hpf)	5	9	6–35	9	8	1–35		
Urine erythrocyte count (/hpf)	4	12	4–25	9	10	1–35		

^a^HPS, hantavirus pulmonary syndrome; ALT, alanine aminotransferase; AST, aspartate aminotransferase; BUN, blood urea nitrogen; CPK, creatinine phosphokinase; LDH, lactate dehydrogenase; PT, prothrombin time; PTT, partial thromboplastin time; hpf, high power field. ^b^There was only one case for this laboratory category.

Three of nine patients had aspartate aminotransferase >500 U/L, alanine aminotransferase >300 U/L, total bilirubin >1.5 mg/dL, and direct bilirubin >1.0 mg/dL. Two patients had clinical evidence of liver disease—one with hepatomegaly and the other with scleral icterus. Hematemesis, melena, and coagulopathy (prothrombin time = 22 s, partial thromboplastin time = 167 s, fibrinogen = 161 mg/dL) also developed in one patient. Two of the three patients also had elevated serum amylase values (294 and 437 U/dL).

Seven of nine patients had chest x-ray examinations on admission. All had evidence of bilateral infiltrates, and one of seven had a radiographic pattern suggestive of pulmonary edema. Two patients who did not have chest x-ray examinations on admission subsequently were found to have bilateral interstitial infiltrates radiographically. Pulmonary edema of varying severity developed in four patients during the course of their illnesses; pleural effusions also occurred in three of these patients, and they were intubated within 24 to 72 hours after admission.

### Community Survey

Interviews and serum specimens were obtained from 311 (83%) of 376 residents of seven different neighborhoods of Las Tablas and Guarare Districts in which confirmed HPS patients were identified. These 311 survey participants represented 119 households. A minimum of one blood specimen was obtained from each household. Forty (13%) of 311 survey participants had IgG against hantavirus. By sex, 25 (14%) of 178 females and 15 (11%) of 133 males had IgG antibodies. The median age of the 40 antibody-positive participants was 31.5 years (range 1–79). Age group prevalence estimates ranged from 9% (5/53, >61years of age) to 21% (6/28, 51–60 years of age). Of 47 children, 5 (11%) <10 years of age had IgG antibodies. Antibody prevalences among patients' neighborhoods ranged from 6% to 31% (Table 4). Two of 10 household members of confirmed patients had IgG antibodies. Six (15%) of the 40 infected participants had visited a confirmed HPS patient.

**Table 4:** Hantavirus IgG antibody-positive findings of survey participants and households by neighborhood, town, and district of confirmed HPS patients, Los Santos Province^a^

Neighborhood (case no.)	Town	District	No. of persons tested	No. (%) persons antibody positive	No. of households tested	No. (%) households antibody positive
2	Los Angeles	Las Tablas	45	4 (8.9)	18	4 (22.2)
4	San Jose	Las Tablas	34	5 (14.7)	11	3 (27.3)
7	Bella Vista	Guarare	50	3 (6.0)	24	2 (8.3)
8	San Jose	Las Tablas	48	15 (31.3)	15	11 (73.3)
9	Santo Domingo	Las Tablas	43	6 (14.0)	15	4 (26.7)
11	Las Tablas	Las Tablas	41	4 (9.8)	15	4 (26.7)
12	El Cocal	Las Tablas	50	3 (6.0)	21	3 (14.3)
Total			311	40 (12.9)	119	31 (26.1)

^a^IgG, immunoglobulin G; HPS, hantavirus pulmonary syndrome.

Of 119 households surveyed, 31 (26%) had at least one member who had IgG against hantavirus (Table 4). Eight (8%) of 96 households had two or more antibody-positive members, while 1 (2%) of 54 households had three or more antibody-positive members. Prevalence of antibody-positive households (i.e., one or more antibody-positive members) per neighborhood ranged from 8% to 73%.

Among the 40 antibody-positive participants, occupations of those with the highest antibody prevalences were students (14%), secretaries (13%), agricultural workers (13%), livestock or vegetable farmers (12%), and housewives (11%). Among the 40 infected participants, 33 (82.5%) touched rodents, 31 (77.5%) cleaned up rodent droppings (e.g., sweeping, mopping), and 29 (72.5%) killed rodents after December 1, 1999. Fifteen (37.5%) noted increased numbers of peridomestic rodents compared with previous years.

Only five (12.5%) of 40 antibody-positive participants recalled fever or myalgia since December 1, 1999. In contrast, the most common symptoms reported were upper respiratory in nature, such as rhinorrhea (45%), sore throat (35%), and cough (22%). Moreover, of two participants who had both IgM and IgG antibodies, one experienced only a cough without fever, while the other was asymptomatic.

### Hospital Serosurvey

Questionnaires and serum samples were obtained from 38 directly exposed and 39 unexposed healthcare workers. No IgM antibodies were present in the 77 workers. Only one of 38 exposed workers had IgG antibodies. This person, a medical resident, directly cared for five confirmed HPS patients (each healthcare worker cared for an average of 4 patients, range 1–5) for a total of approximately 15 patient-care days (group mean 43, range 1–70). His first exposure occurred in late December 1999. While caring for patients, he wore gloves, gown, and mask most of the time and was not directly exposed to respiratory secretions. He denied any travel to Los Santos Province after December 1999 and had no history of exposure to rodents. However, he was uncertain about whether he had visited Los Santos Province before December 1999. He denied having any febrile illness after late December 1999. Of the remaining 37 healthcare workers who cared for HPS patients, four (11%) were directly exposed to respiratory secretions in the eye, nose, or mouth; 26 (70%) wore gloves, gowns, and masks most of the time.

One unexposed worker, an operating room assistant, also had IgG against hantavirus. She denied travel to Los Santos Province, exposure to rodents, or febrile illness after December 1999. However, history of travel to Los Santos Province before December 1999 was again uncertain.

### Rodent Investigation

Rodent traps were set at 13 sites, 10 of which were homes and immediate surroundings of patients with confirmed and suspected HPS. One of the three remaining sites was the home of a patient previously suspected to have HPS who did not have antibodies to hantavirus. The other two locations were a rural agricultural area in the Pocri District of Los Santos Province and a late secondary forest area (two subsites) near the town of Portabelo, Montijo District, Veraguas Province. In all, 120 rodents representing nine species and seven opossums (two species) were captured (Table 5). Of the 120 rodents, 52 (43%) were caught from the 10 patient household areas. A trap success of approximately 5% was achieved at these homes. Only one of the six antibody-positive rodents (*Oligoryzomy fulvescens*) was captured at the household of a confirmed HPS case-patient (Las Tablas town); five were captured in the Pocri District.

**Table 5:** List of species of rodents and opossums captured at 13 trapping sites in Los Santos and Veraguas Provinces, Panama, February–March 2000, and antibody results

Rodent species (common name)	No. captured	No. hantavirus antibody-positive
*Zygodontomys brevicauda cherriei* (cane rat)	50	4
*Sigmodon hispidus* (hispid cotton rat)	20	0
*Oligoryzomys fulvescens* (fulvous pigmy rice rat)	15	2
*Oryzomys couesi* (Coues's Rice rat)	14	0
*Mus musculus* (house mouse)	9	0
*Liomys adspersus* (Panamanian spiny pocket mouse)	8	0
*Oryzomys concolor* (colored rice rat)	2	0
*Proechimys semispinosus* (silky spiny rat)	1	0
*Rattus rattus* (black rat)	1	0
*Marmosa* *mexicana*. (Mexican mouse opossum)	4	0
*Marmosa robinsoni* (Robinson's mouse opossum)	3	0

## Discussion

We have documented the first human cases of hantavirus infection in Central America. A novel Panamanian hantavirus, Choclo virus, was subsequently characterized and is thought to be responsible for HPS during the outbreak (*33*). Virus genetic sequences of Choclo virus from case-patients were identical to those from *O. fulvescens* (*33*).

The clinical spectrum of pulmonary disease among HPS patients in Panama varied widely from severe disease requiring intubation and cardiovascular support to mild pulmonary involvement with a benign clinical course. Extrapulmonary manifestations, such as hepatobiliary disease, hemorrhage, and central nervous system sequelae, were also present. The case-fatality rate (0% among 9 confirmed cases; 25% among 12 total suspected and confirmed cases) was noticeably lower than that described in Paraguay (12%), Chile (54%), and the United States (52%) in confirmed cases (*5**,**9**,**29*).

We found an antibody prevalence of 13% among household and neighborhood members of all ages from the outbreak foci. This figure is comparable to that found in Paraguay, but higher than in Chile and the United States (*9**,**29**,**36*). The percentages of hantavirus antibody-positive persons and households were particularly high in San Jose, the only town with two confirmed case-patients. Clustering of cases of HPS was not observed, and household clustering of antibody-positive persons was infrequent. Only 13% of hantavirus antibody-positive persons had a febrile illness after early December 1999, and none had an illness compatible with HPS, which suggests that mild infections occurred, as documented with other hantaviruses in the United States and Chile (*9**,**36**–**38*).

Person-to-person transmission of hantavirus infection was not demonstrated during the outbreak. Infrequent clustering of antibody-positive serosurvey participants by household probably reflected common exposures to infected rodent excreta peridomestically. Furthermore, only 1 of 38 medical care workers who cared for HPS patients had IgG antibodies, while none had IgM antibodies. Similarly, 1 of 39 workers who did not care for HPS patients had IgG antibodies. Thus far, person-to-person transmission has only been suggested during outbreaks caused by Andes virus in Argentina and Chile (*7**–**9*).

**Figure 2:**
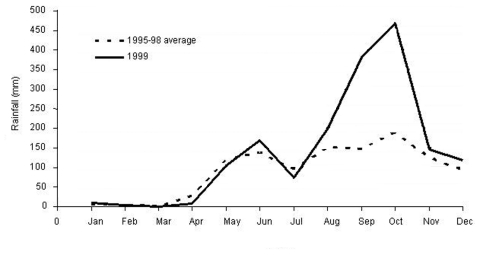
Comparison of monthly rainfall in 1999 with the average monthly rainfall from 1995 to 1998, Los Santos Province, Panama.

Climate data from Los Santos Province clearly demonstrated a two- to threefold increase in rainfall in September and October 1999 when compared to similar periods in the previous 4 years (Figure 2). Such atypical rainfall patterns may have led to increases in rodent populations, which led to more frequent contact between infected rodents and humans and subsequent human infection. Many residents of patient-neighborhoods of Las Tablas reported an unusually large number of rodents from December 1999 through January 2000. Similar patterns of environmental change followed by outbreaks of human disease have been observed in the United States and South America (*29**,**39**,**40*).

Ecologic observations in Los Santos provided an impression of a dry, open, and deforested region. Long-term commercial logging, agriculture (primarily corn and sugarcane), and animal husbandry practices (mainly cattle and horses) have contributed to the deforestation process. The rodent species most closely associated with corn and sugarcane fields, *Zygodontomys brevicauda cherriei*, is a likely reservoir host for a new hantavirus, Calabazo virus (*33*). *O. fulvescens*, the likely reservoir for Choclo virus, was found in grass of varying heights near human habitation and in cattle and horse pastures. Given the observed habitat associations of these two species, agriculture and animal husbandry practices in the Los Santos regions may have had a positive effect on populations of rodents associated with hantaviruses and may continue to augment the risk for HPS as the human population increases.

The social and economic impact of this outbreak was substantial. The cancellation of Carnival, one of Panama's most celebrated festivals, had financial effects on residents of Las Tablas. Nevertheless, the public health impact of holding Carnival could also have been substantial, given the potential for rodent exposure among thousands of visitors and participants.

In conclusion, this outbreak resulted in the first documented cases of human hantavirus infections in Central America. Although cases were not reported from other districts of Los Santos or other provinces of Panama during the investigation, surveillance for HPS nationwide should continue, as serologic testing capabilities have since been implemented in Panama. More extensive sampling of rodent populations would help identify other areas in Panama with large numbers of *O. fulvescens* that could place residents at risk for Choclo virus infection. Longitudinal studies of rodents, particularly those species implicated as reservoir hosts, will be necessary to monitor the fluctuation and distribution of rodent population numbers over time and their correlation with human infection (*41*). Educational campaigns promoting risk reduction, such as proper clean-up of rodent excrement, sealing homes against the entry of rodents, and other rodent-proofing techniques, should continue as additional cases of HPS are reported in Panama.
